# A feasibility randomised trial comparing therapeutic thoracentesis to chest tube insertion for the management of pleural infection: results from the ACTion trial

**DOI:** 10.1186/s12890-022-02126-4

**Published:** 2022-08-30

**Authors:** David T. Arnold, Emma Tucker, Anna Morley, Alice Milne, Louise Stadon, Sonia Patole, George W. Nava, Steven P. Walker, Nick A. Maskell

**Affiliations:** 1 Academic Respiratory Unit, University of Bristol, Level 2, Learning & Research Building, Southmead Hospital, Bristol, BS10 5NB UK; 2 North Bristol NHS Trust, Bristol, UK

**Keywords:** Pleural empyema, Pleural effusion, Parapneumonic

## Abstract

**Background:**

Pleural infection is a complex condition with a considerable healthcare burden. The average hospital stay for pleural infection is 14 days. Current standard of care defaults to chest tube insertion and intravenous antibiotics. There have been no randomised trials on the use of therapeutic thoracentesis (TT) for pleural fluid drainage in pleural infection.

**Aims and objectives:**

To assess the feasibility of a full-scale trial of chest tube vs TT for pleural infection in a single UK centre. The primary outcome was defined as the acceptability of randomisation to patients.

**Methods:**

Adult patients admitted with a pleural effusion felt to be related to infection and meeting criteria for drainage (based on international guidelines) were eligible for randomisation. Participants were randomised (1:1) to chest tube insertion or TT with daily review assessing need for further drainages or other therapies. Neither participant nor clinician were blinded to treatment allocation. Patients were followed up at 90 days post-randomisation.

**Results:**

From September 2019 to June 2021, 51 patients were diagnosed with pleural infection (complex parapneumonic effusion/empyema). Eleven patients met the inclusion criteria for trial and 10 patients were randomised (91%). The COVID-19 pandemic had a substantial impact on recruitment. Data completeness was high in both groups with no protocol deviations. Patients randomised to TT had a significantly shorter overall mean hospital stay (5.4 days, SD 5.1) compared to the chest tube control group (13 days, SD 6.0), *p* = 0.04. Total number of pleural procedures required per patient were similar, 1.2 in chest tube group and 1.4 in TT group. No patient required a surgical referral. Adverse events were similar between the groups with no readmissions related to pleural infection.

**Conclusions:**

The ACTion trial met its pre-specified feasibility criteria for patient acceptability but other issues around feasibility of a full-scale trial remain. From the results available the hypothesis that TT can reduce length of stay in pleural infection should be explored further.

*Trial registration*: ISRCTN: 84674413.

## Background

Pleural infection is a complex condition with a considerable healthcare burden. The current standard of care for pleural infection is prompt pleural fluid drainage and antibiotics [1–3]. Patients are usually admitted to hospital for chest tube placement and intravenous antibiotics. Although some patients will require intrapleural fibrinolytics or thoracic surgery for resolution, the majority will resolve with pleural fluid drainage alone. Despite this the average hospital length of stay for pleural infection is 14 days, placing a considerable burden on the patient, their families and health services [4].

One of the potential barriers to hospital discharge in pleural infection is the insertion of a chest tube which can only be managed in hospital, causes discomfort and reduces ambulation. Numerous studies have shown that reduced ambulation has a significant impact on recovery and rehabilitation [5]. Ambulatory strategies for pleural fluid drainage have been highly successful in reducing hospital stay for malignant pleural effusions [6]. Similar management approaches have never been prospectively trialled for pleural infection.

Therapeutic thoracentesis (TT) is an alternative to chest tube insertion that confers some theoretical advantages. TT involves the insertion of a smaller temporary catheter into the infected pleural space under ultrasound guidance. Pleural fluid is then aspirated and the catheter removed [7]. Not only is the patient able to mobilise after, TT is a simpler and quicker procedure, reduces the risk of site infection and bleeding, and can be better directed at multi-loculated effusions. In addition, TT removes the risk of accidental chest tube displacement, which affects up to 30% of cases [8], or retained guidewires [9]. However, there is a potential requirement for repeated procedures on patients, with cumulative exposure to procedural complications. It is also uncertain whether continuous drainage via a standard chest tube offers additional benefit, in terms of time to resolution, compared with repeated TT.

There are no randomised trials comparing TT to chest tube drainage in pleural infection.

## Methods

### Aim

To assess the feasibility of a randomised trial of chest tube versus therapeutic thoracentesis in pleural infection.

### Design

Single centre randomised feasibility trial of chest tube versus therapeutic thoracentesis in patients presenting to secondary care with proven pleural infection. The target population for this feasibility study are patients who have been diagnosed with pleural infection requiring drainage based on well recognised criteria (see Inclusion Criteria). These patients would normally be managed with a chest tube and hospital admission as per current guidance [2, 3].

### Participants

We included adult patients (≥ 18 years old) with a clinical presentation consistent with pleural infection and fulfilling at least one of the following criteria; purulent pleural fluid, pleural fluid pH ≤ 7.2, pleural fluid glucose ≤ 3.4 mmol/L, pleural fluid LDH > 1000 IU, pleural fluid Gram stain and/or culture positive for bacteria, or a large effusion occupying > 50% of hemithorax.

We excluded those with severe septations/loculations on pleural ultrasound precluding chest tube insertion, with signs of ongoing sepsis requiring support beyond basic fluid resuscitation with uncorrectable coagulopathy or those unable to consent for study. Those with a previous pneumonectomy, recent thoracic surgery or indwelling pleural catheter in situ were also excluded. Given the potential need for regular unplanned outpatient appointments in the intervention group it was not felt possible to randomised patients currently in prison.

The RAPID score is a validated prognostic tool that can predict a patient’s risk of mortality from pleural infection [10]. A high RAPID score, inferring a higher risk of mortality at 3 months, was originally included as an exclusion criterion. However, the decision was made to remove this criterion by the trial management group after 2 patients who appeared otherwise appropriate for trial inclusion were excluded based on RAPID score alone. Whilst the score is a useful indicator of mortality it has not been validated to dictate management.

### Outcome measures

The primary outcome of the Aspiration versus Chest Tube drainage for pleural infectION (ACTion trial) was feasibility, with a focus on the acceptability of the trial intervention to patients. This was defined *ad priori* as the proportion of the total number of patients who are eligible for trial entry that accepted randomisation. The primary outcome was considered successful if ≥ 50% of eligible patients were willing to be randomised.

Secondary outcomes included the (i) number of pleural interventions, (ii) use of intrapleural fibrinolytics and referrals for surgical intervention, (iii) the patients’ hospital length of stay and duration of intravenous antibiotic therapy (iv) readmission rates, (v) patient-reported outcomes measures (PROMS), including health related quality of life (HRQoL), using the EQ-5D-5L questionnaire [11] and Visual Analogue Score (VAS) for common symptoms of pleural infection; chest pain and shortness of breath.

All exploratory secondary outcomes measures have been chosen as they are clinically relevant and present valid options for the primary outcome measure for a subsequent full-scale trial.

### Screening and randomisation

Patients with suspected pleural infection were screened for eligibility by a member of the trial team. A screening log was kept to document reasons for screen failure. Eligible patients were approached by a member of the clinical trial team and given a trial information sheet (TIS). Following consent, randomisation was performed using a secure web-based system (REDCap). Participants were randomised in a 1:1 ratio to either the control (chest tube) or interventional (TT) arm of the study. It was impractical to conceal treatments from either patients or healthcare professionals.

### Post randomisation

#### Control arm (chest tube)

Patients allocated to the control arm had a chest tube inserted as per standard care, with minimal delay between randomisation and intervention in keeping with usual care for urgent pleural drainage. The size of chest tube was left to the discretion of the treating physician and a Seldinger technique was used for tube insertion after bedside ultrasound marking. The chest tube was attached to an underwater seal and drained as per local hospital policy. Any chest tube blockages were managed with a small volume flush of 10-20 ml of normal saline.

#### Intervention arm (TT)

Patients allocated to the intervention arm had a TT performed. Drainage was performed using a 6Fr or 8Fr Rocket thoracentesis kit after bedside ultrasound marking.

The effusion was drained until drainage stopped or until the patient developed symptoms associated with trapped lung (coughing or chest pain). Any catheter blockages were managed with a small volume flush of 10–20 ml of normal saline.

### Post procedure inpatient period

Whilst in hospital, all patients had a clinical review at least every working day. As a minimum a chest radiograph and thoracic ultrasound was performed on Day 3 (± 1 day) and Day 7 (± 1 day), with additional imaging if deemed necessary by the treating physician. Blood tests were performed on at least alternate days until Day 7, and then at least weekly if the patient remained in hospital. Antibiotic use and discharge from hospital was left to the discretion of the treating physician.

### Repeated pleural procedures or rescue therapies (intrapleural fibrinolytics or surgery)

The requirement for additional pleural procedures or rescue therapies was at the discretion of the treating clinician. The protocol recommended they consider clinical/biochemical factors (such as fevers or rising C-Reactive Protein (CRP)/White Blood Cell count), chest radiograph and pleural ultrasound appearances suggesting persisting collections, when making a decision.

### Trial follow-up

Patients had a trial review at Day 3 and Day 7, and in outpatient clinic at Day 90 post randomisation (± 7 days) where a chest X-ray, ultrasound and clinical review (including bloods and PROMS) were performed.

### Sample size

As a feasibility study, sample sizes were not formally calculated although it was estimated that 30 patients randomised (15 in each arm) would be achievable in the time frame.

### Statistical analysis

Quantitative analysis was focused on obtaining estimates and measures of variation of key unknowns required for the design of a full-scale randomised trial of TT versus conventional chest tube in pleural infection. Specifically:

- Descriptive statistics for rates of recruitment, randomisation, attrition and data completion (including differences between the intervention and controls).
- Descriptive statistics for the proposed secondary outcomes (including hospital length of stay, number of procedures, readmission rates, intravenous antibiotic use, fibrinolytic use and surgical referrals), and common adverse clinical events (such as persistent fever, or abnormal blood chemistry and radiography).

### Impact of the COVID-19 pandemic

In March 2020, the ACTion trial was paused, alongside all non-COVID-19 research at the recruiting centre. After trial resumption in August 2020, it was apparent that the pandemic had fundamentally changed the presentation of respiratory infection. This has particularly affected influenza and pneumococcal transmission which contributes to pleural infection rates [12, 13], especially in the young who are most likely to be eligible for the ACTion trial. An application for an extension to the ACTion trial was made in January 2021 in order to maximise recruitment. The trial duration was subsequently extended by 12 months.

## Results

From September 2019 to June 2021, 51 patients were identified with pleural infection. Of the 11 patients who met the inclusion criteria for trial, 10 patients were randomised (91% C.I. 0.58–0.99). Prior to the COVID-19 pandemic, the trial was recruiting to time and target. The pandemic had a significant impact on pleural infection admissions and research resource allocation, as described in the Methods section.

Review of screening logs provided further information about the recruitment. Of the 51 patients that met the criteria for pleural infection, 24 had an exclusion criterion and from the remaining 16 that were potentially eligible for the trial only 11 were given a Trial Information Sheet. See Table 1 for screening log summary. The most common reason for exclusion was a lack of capacity to consent for the study (7 related to permanent memory impairment/dementia and 5 relating to transient delirium).

**Table 1:** ACTion trial screening summary

Total screen positive (proven pleural infection)	51
*Not approached for randomisation*	40
Met inclusion criteria but excluded for:	
Heavily loculated (unable to drain)	6
Lacks capacity	12
< 18yo	1
High RAPID score	2
Prisoner	3
Deemed too unwell by treating team	6
Recruited to another interventional trial	3
Study team not informed of patient prior to pleural intervention	5
COVID-19 pandemic pause on recruitment	2
*Total approached for randomisation*	11
Randomised	10
Patient declined trial entry	1

### Data completeness

The Baseline Case Report Form (CRF) was completed in full for all participants including HRQoL metrics. Day 3 CRF completeness had a single missing value from 1 variable as an albumin was not performed for a patient whilst an inpatient. Day 7 CRF was completed in full for all participants. Day 90 CRF was completed in full for all participants.

### Clinical outcomes

#### Baseline demographics

The cohort had an average age of 68 and a male predominance. There were no patients with a WHO performance status above 2. The majority of patients fell into the ‘Medium’ risk RAPID score category, see Table 2.

**Table 2:** Demographics of participants by intervention

	Chest tube (n = 5)	Therapeutic thoracentesis (n = 5)	All (n = 10)
Age (range)	73 (57–87)	62 (45–77)	68 (45–87)
Male	3 (60%)	3 (60%)	6 (60%)
Recruited as IP/OP	4/1	4/1	8/2
Active malignancy?	0	1	1
Major comorbidities			
Cardiac	1	1	2
Respiratory	1	1	2
Liver	0	0	0
Renal	0	1	1
Alcohol consumption > 20units weekly	1	2	3
Active or previous IVDU	1	0	1
Current or ex-smoker	2	4	6
RAPID score			
Low	0	0	0
Medium	4	5	9
High	1	0	1

*IP* inpatient, *OP* outpatient, *IVDU* intravenous drug use

All patients had community-acquired infections with the majority having felt unwell for at least 2–4 weeks prior to admission. Given the small numbers, formal comparisons of baseline characteristics have not been performed, see Table 3.

**Table 3:** Details of pleural infection by intervention

	Chest tube (n = 5)	Therapeutic thoracentesis (n = 5)	Total cohort (n = 10)
Community acquired (%)	5 (100%)	5 (100%)	10 (100%)
Mean duration of symptoms in weeks (range)	3 (1–6)	3 (1–8)	3 (1–8)
Right sided	3 (60%)	0 (0%)	3 (30%)
Size on chest radiograph			
Small (< 25%)	0	2	2
Moderate (25–49%)	4	3	7
Large (≥ 50%)	1	0	1
Degree of complexity on pleural ultrasound			
None	0	2	2
Mild	4	3	7
Moderate	1	0	1
Heavy	0	0	0
Pleural thickening on ultrasound	4	3	7
*Pleural fluid analysis (mean, SD)*			
pH	7.0 (0.2)	7.0 (0.1)	7.0 (0.2)
LDH (iu/L)	1655 (1886)	1357 (608)	1506 (1330)
Glucose (mmol/L)	0.4 (1.2)	0.7 (1.4)	0.5 (1.2)
Gram stain positive	0	0	0
Culture positive	1- Staphylococcus Intermedius 1- Staphylococcus aureus	0	2

*SD* Standard Deviation, *CRP* C Reactive Protein, *LDH* Lactate Dehydrogenase

#### Hospital length of stay and intravenous antibiotic duration

The mean overall length of stay in hospital was 9.2 (SD 6.6) days, see Table 4. On average, the diagnosis of pleural infection was made on day 3 of the hospital admission, with a length of stay post-diagnosis (and randomisation) of 7.7 days. Patients randomised to TT had a significantly shorter overall mean hospital stay (5.4 days, SD 5.1) compared to the chest tube control group (13.0 days, SD 6.0), *p* = 0.04.

**Table 4:** Management outcomes

	Chest tube (n = 5)	Therapeutic thoracentesis (n = 5)
Mean hospital length of stay in days (mean, SD)	13.0 (6.0)	5.4 (5.1)
Diagnosis to discharge in days (mean, SD)	10.4 (4.8)	5.0 (5.0)
Readmission within 90 days	0	1
Number of outpatient clinic appointments	3	6
Mean intravenous antibiotic use in days (mean, SD)	9.8 (4.3)	4.6 (4.3)
Total number of pleural procedures	6 (1.2)	7 (1.4)
Total number of TTs performed (per patient)	0 (0)	6 (1.2)
Total number of chest tubes performed (average)	6 (1.2)	1 (0.2)
Duration of chest tube in-situ in days (mean, SD)	6.8 (3.9)	N/A
Courses of fibrinolytics	3	1
Saline irrigation performed	0	0
Referral for thoracic surgery	0	0
Mean quantity of pleural fluid drained in mls (SD)	1366 (1369)	1034 (994)
Adverse events	1	1
Serious adverse events	0	1

*SD* Standard deviation

For all patients the mean intravenous antibiotic duration was 8.1 days. Patients randomised to TT had a shorter median antibiotic duration (4.1 days, SD 3.9) compared to the chest tube control group (11.0 days, SD 4.1), *p* = 0.03.

#### Adverse events and readmissions

There were 3 AEs recorded during the trial period. One patient in the TT group had an SAE recorded as they were readmitted to hospital within the Day 90 follow-up period, for a pre-existing medical problem that was not related to the trial intervention or pleural infection. A patient in the chest tube group had an expected AE where the chest tube became dislodged and required an attempted reinsertion on the ward. Finally, a patient in the TT group continued to spike fevers over 38 degrees 24 h after the initial TT was performed. These were documented as expected AEs as per protocol. It was decided due to rapid loculation of the pleural effusion to insert a chest drain for further fluid management.

#### Number of procedures and use of fibrinolytics

Amongst the 10 patients, 13 pleural procedures (excluding the initial diagnostic thoracentesis) were performed from admission to Day 90 follow-up, see Table 4 for full details. The total number of pleural procedures required per patient were similar between groups: 1.2 in chest tube group and 1.4 in TT group.

#### Radiographic outcomes

Patients had a chest radiograph and thoracic ultrasound performed on Day 3 (± 1 day) and Day 7 (± 1 day). These were repeated at Day 90 in outpatient follow-up. One patient did not attend 90 day follow up due to the cancellation of all non-urgent appointments during the COVID-19 pandemic, so did not have an ultrasound, but had blood tests and chest radiograph performed in primary care.

By 90-day follow-up, there had been complete resolution of pleural effusions seen on admission in all but 2 patients, both in the chest tube group, both patients had received intrapleural fibrinolytics.

#### Patient reported outcome measures

**Fig. 1:**
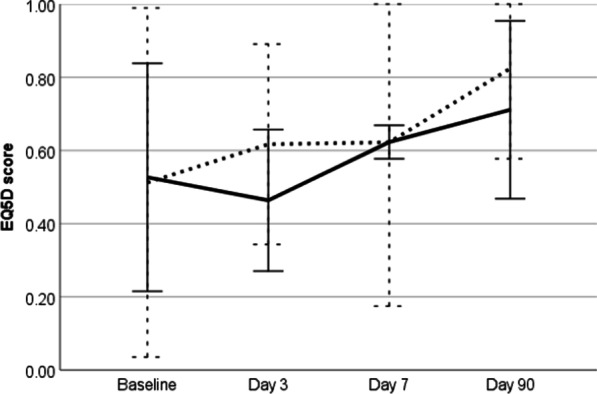
Patient reported EQ-5D scores (mean and 95% confidence intervals) in patients randomised to chest tube (solid line) and therapeutic thoracentesis (dashed line) at each trial visit

Patients rated their overall health negatively at the baseline assessment with a mean EQ5D score of 0.52 (SD 0.31), see Fig. 1. Patients reported higher levels of breathlessness compared to chest pain at baseline with mean values of 40 (SD 27) and 28 (SD 27) on the 0 to 100 scale respectively. The randomised groups were well matched at baseline in term of overall health and VAS scores for breathlessness and chest pain.

Following randomisation and the subsequent pleural procedure, mean symptom scores improved for all patients at Day 3 with VAS breathlessness falling from 40 to 26 and VAS chest pain falling from 28 to 19. Across the entire follow-up period there was no significant difference between the groups in terms of overall EQ-5D scores or individual VAS symptom scores.

## Discussion

This feasibility study has shown that randomising patients with pleural infection to therapeutic thoracentesis or chest tube insertion is, at least in part, feasible. Over 90% of eligible patients offered entry into the trial accepted and were randomised. Additionally, the trial design and processes were acceptable to participants. The intervention has been shown to be safe in previous case series and there were no serious adverse events related to the trial, although absolute numbers were small. From the patients recruited there was a suggestion that hospital length of stay was reduced in the intervention group, a finding that should be explored in a full-scale trial. However, modifications to the intervention and trial procedures are needed before a full-scale trial is undertaken.

This is the first randomised trial comparing TT to the standard of care, chest tube insertion, in pleural infection. A literature review revealed several case series documenting its safe use in European centres [14]. Storm et al. published a retrospective study comparing the outcomes of 94 patients with pleural infection [15]. Over a 5-year period (1984 to 1989), 51 patients were treated with TT in a medical ward and retrospectively compared to 43 patients treated with chest tube who were admitted to a surgical ward. Although the hospital stay was longer than more recent studies (potentially due to more serious infection at baseline and/or differing medical practices), it was considerably shorter in the TT group (2.3 versus 5 weeks). A more recent case series from France retrospectively analysed patients with pleural infection who had been managed with TT over a 9-year period (2001–2010) [16]. The median number of TTs required per patient was 3 (IQR 2–5) with a median hospital length of stay of 21 days. The strategy failed in 15/79 (19%) with 12 patients needing a chest tube inserted and 3 who required thoracic surgery. Importantly, TT complication rates were low despite ultrasound guidance only being used in 53% of the patients. This case series has recently been updated for the period 2011 to 2018 [17]. There is no comment on patient tolerability of the approach and complications are similar to those reported in chest drain literature.

The only prospective study of TT in pleural infection was carried out in a paediatric population with ‘severe empyema’[18] between 1992 and 1999. Shoseyov and colleagues performed a non-randomised study comparing TT to chest tube, by virtue of differing practices between three regional hospitals. TT was carried out on alternate days until clinical or radiographic resolution which took on average 2.4 aspirations per patient (range 1 to 4). They found no significant difference between groups in terms of length of stay (24 days in the chest tube group versus 22 days in the TT group), duration of fever, length of antibiotic therapy, amount fluid drained, or need for thoracic surgery. Given the non-randomised nature of these studies it is impossible to directly compare the two treatment methods, but it does demonstrate that TT was safe, with a suggestion of a reduction in hospital stay.

The ACTion trial was set up to assess the feasibility of a full-scale trial of TT versus chest tube for pleural infection. Although the trial met its pre-specified criteria for feasibility, there are other elements to consider when, or if, a full-scale trial is planned. The ACTion trial shows that the concept of the intervention and other trial protocols (questionnaires, follow-ups, blood tests) are acceptable to patients hospitalised with pleural infection. 91% of patients approached accepted randomisation into the trial and there were no withdrawals post-randomisation. Data completeness was high. However, the slow recruitment is a concern for designing a full-scale trial on the intervention, even in the context of COVID-19, and we would suggest adaptions to the protocol to improve recruitment.

A considerable number of patients were excluded from participating in the trial due to lacking capacity at the time of enrolment. This is likely to be an issue for a full-scale trial and there should be a consideration as to whether deferred or personal consultee consent would be appropriate. Patients without capacity secondary to delirium or dementia are a population who are likely to suffer adverse events from chest tube insertion and therefore might benefit most from this trial.

Patient acceptability of randomisation has been demonstrated but acceptability to physicians and other healthcare professionals remains uncertain. Six potentially eligible patients were not offered randomisation on the basis of “being too unwell” without meeting the exclusion criterion of “signs of ongoing sepsis requiring support beyond basis fluid resuscitation”. Given that this trial was run in a unit with experience running interventional pleural studies it can be extrapolated that physician withdrawal would be greater at other hospital sites in a multicentre study. There is no evidence that TT is an inadequate method of source control compared to chest tube. It is crucial that the physicians carrying out the full-scale trial have equipoise and are comfortable with the intervention.

This feasibility trial has weaknesses that affect the generalisability of its findings. Firstly, just 10 patients were recruited so outcome data should be interpreted with great caution. Had the trial met its pre-specified target of 30 patients the additional data generated would have increased confidence in the feasibility of a full-scale trial and might have raised further issues that could be addressed for future studies. The drop in community transmitted bacterial infections, especially pneumococcal, and possible change in healthcare-seeking behaviour caused a significant drop in pleural infection incidence during the trial recruitment period [19]. Secondly, as a single centre trial, issues might have been missed that might have been realised in a multicentre study. However, the presentation and diagnosis of pleural infection does not differ significantly by centre, and the interventions (chest tube and therapeutic thoracentesis) are basic procedural skills for acute medical and respiratory physicians.

## Conclusions

This is the first randomised trial of chest tube versus TT for patients presenting with pleural infection. The trial processes and intervention appear acceptable to patients, and a full-scale trial is feasible. Such a trial should explore the suggestion of a reduced hospital length of stay in patients initially managed with therapeutic thoracentesis.

## Data Availability

All data generated or analysed during this study are included in this published article.

## Abbreviations

ACTion: Aspiration versus chest tube drainage for pleural infectION
AE: Adverse event
C.I.: Confidence interval
CRF: Case report form
CRP: C Reactive protein
HRQoL: Health related quality of life
LDH: Lactate dehydrogenase
SD: Standard deviation
VAS: Visual analogue score
WHO: World Health Organisation

## References

[1] Colice GL, Curtis A, Deslauriers J, Heffner J, Light R, Littenberg B (2000). Medical and surgical treatment of parapneumonic effusions : an evidence-based guideline. Chest.

[2] Davies HE, Davies RJ, Davies CW, Group BTSPDG. Management of pleural infection in adults: British Thoracic Society Pleural Disease Guideline 2010. Thorax. 2010;65 Suppl 2:ii41–53. 10.1136/thx.2010.137000 20696693

[3] Shen KR, Bribriesco A, Crabtree T, Denlinger C, Eby J, Eiken P (2017). The American Association for Thoracic Surgery consensus guidelines for the management of empyema. J Thorac Cardiovasc Surg.

[4] Arnold DT, Hamilton FW, Morris TT, Suri T, Morley A, Frost V (2021). Epidemiology of pleural empyema in English hospitals and the impact of influenza. Eur Respir J..

[5] Mundy LM, Leet TL, Darst K, Schnitzler MA, Dunagan WC (2003). Early mobilization of patients hospitalized with community-acquired pneumonia. Chest.

[6] Roberts ME, Neville E, Berrisford RG, Antunes G, Ali NJ, Group BTSPDG. Management of a malignant pleural effusion: British Thoracic Society Pleural Disease Guideline 2010. Thorax. 2010;65(Suppl 2):ii32–40. 10.1136/thx.2010.136994 20696691

[7] Jouneau S, Letheulle J, Desrues B (2015). Repeated therapeutic thoracentesis to manage complicated parapneumonic effusions. Curr Opin Pulm Med.

[8] Rahman NM, Pepperell J, Rehal S, Saba T, Tang A, Ali N (2015). Effect of opioids vs NSAIDs and larger vs smaller chest tube size on pain control and pleurodesis efficacy among patients with malignant pleural effusion: the TIME1 randomized clinical trial. JAMA.

[9] Harris A, O'Driscoll BR, Turkington PM (2010). Survey of major complications of intercostal chest drain insertion in the UK. Postgrad Med J.

[10] Rahman NM, Kahan BC, Miller RF, Gleeson FV, Nunn AJ, Maskell NA (2014). A clinical score (RAPID) to identify those at risk for poor outcome at presentation in patients with pleural infection. Chest.

[11] Herdman M, Gudex C, Lloyd A, Janssen M, Kind P, Parkin D (2011). Development and preliminary testing of the new five-level version of EQ-5D (EQ-5D-5L). Qual Life Res.

[12] Amin-Chowdhury Z, Aiano F, Mensah A, Sheppard CL, Litt D, Fry NK (2021). Impact of the coronavirus disease 2019 (COVID-19) pandemic on invasive pneumococcal disease and risk of pneumococcal coinfection with severe acute respiratory syndrome coronavirus 2 (SARS-CoV-2): prospective national cohort study. England Clin Infect Dis.

[13] Feng L, Zhang T, Wang Q, Xie Y, Peng Z, Zheng J (2021). Impact of COVID-19 outbreaks and interventions on influenza in China and the United States. Nat Commun.

[14] Porcel JM (2017). Minimally invasive treatment of complicated parapneumonic effusions and empyemas in adults. Clin Respir J..

[15] Storm HK, Krasnik M, Bang K, Frimodt-Moller N (1992). Treatment of pleural empyema secondary to pneumonia: thoracocentesis regimen versus tube drainage. Thorax.

[16] Letheulle J, Tattevin P, Saunders L, Kerjouan M, Lena H, Desrues B (2014). Iterative thoracentesis as first-line treatment of complicated parapneumonic effusion. PLoS ONE.

[17] Luque Paz D, Bayeh B, Chauvin P, Poizeau F, Lederlin M, Kerjouan M (2021). Intrapleural use of urokinase and DNase in pleural infections managed with repeated thoracentesis: a comparative cohort study. PLoS ONE.

[18] Shoseyov D, Bibi H, Shatzberg G, Klar A, Akerman J, Hurvitz H (2002). Short-term course and outcome of treatments of pleural empyema in pediatric patients: repeated ultrasound-guided needle thoracocentesis vs chest tube drainage. Chest.

[19] Ur Rehman K, Bedawi EO, Sivakumar P, Ferguson K, Ajmal S, Graham E (2021). Impact of the COVID -19 pandemic on pleural infections: a multicentre retrospective analysis. Eur Respir J.

